# Aberrant hepatic artery in an elderly patient with pancreatic head tumor

**DOI:** 10.1016/j.sopen.2023.09.011

**Published:** 2023-09-22

**Authors:** Giovanna Di Meo, Piercarmine Panzera, Alessandro Pasculli, Francesco Paolo Prete, Angela Gurrado, Mario Testini

**Affiliations:** Department of Precision and Regenerative Medicine and Ionian Area, University of Bari “Aldo Moro”, Bari, Italy

**Keywords:** Aberrant artery, Hepatic artery, Pancreas cancer, Pancreas surgery, Vascular abnormalities

An 80-year-old woman with a previous hysterectomy performed to address uterine fibroids, and a history of hypothyroidism, without any family history of pancreatic disease, was admitted to our department. She presented with persistent abdominal pain and obstructive jaundice for the past four weeks. Relevant laboratory results showed elevated levels of aspartate transaminase (244 U/L), alanine transaminase (339 U/L), gamma-glutamyl transpeptidase (774 U/L), total bilirubin (1.80 mg/dL), and a CA 19.9 level of 111 U/mL. A tumor of the pancreatic head and uncinate process was suspected on the basis of enhanced computed tomography (CT) findings. Moreover, the CT scan showed an unusual vascular anatomical variant of the hepatic artery (HA) running through the pancreatic parenchyma [1,2] (Fig. 1). A clear tissue plane between the tumor and the aberrant HA could still be found. Prior to the operation, a thoracic CT scan was conducted, revealing no signs of lung metastasis. The MRI demonstrated a dilated common bile duct, measuring approximately 13 mm, with a sudden change in diameter in its distal section and a 17 mm long stricture. A solid mass of around 20 mm, causing biliary stenosis, was observed, displaying focal enhancement after contrast administration and restricted signal in DWI sequences. Consequently, the patient underwent endoscopic retrograde cholangiopancreatography (ERCP), and a biliary stent was placed. During the operation, as there was no definite evidence of arterial invasion, the aberrant HA was meticulously isolated and dissected from the surrounding pancreatic parenchyma (Fig. 2). Therefore, the patient underwent a pancreaticoduodenectomy with preservation of the anomalous HA. What is the most likely aberration of the HA based on these CT and intra-operative image?

1. Michels type IX/Hiatt type V
2. Michels type III/Hiatt type III
3. Michels type IV/Hiatt type IV
4. Michels type VI/Hiatt type III.

**Fig. 1:**
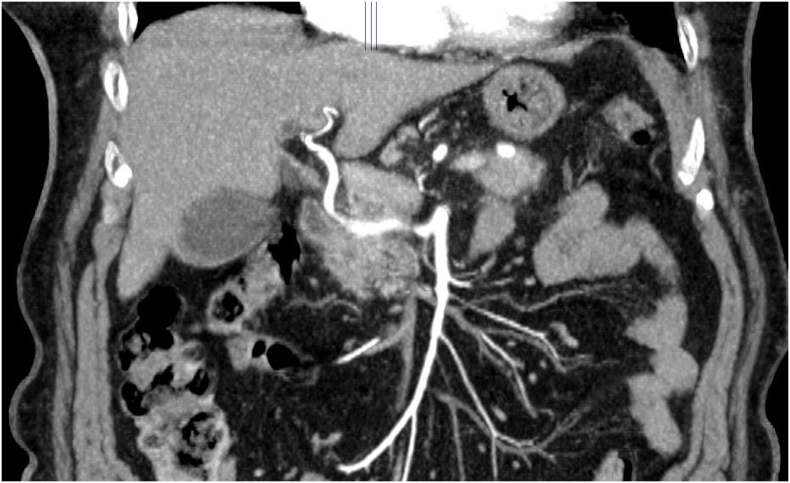
CT image shows the replaced CHA.

**Fig. 2:**
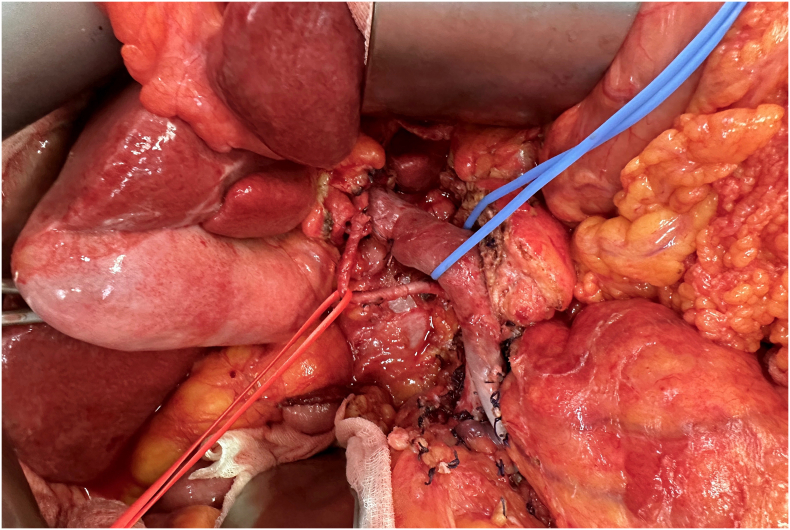
Preservation of the aberrant CHA: intraoperative view.

## Funding sources

This article did not receive any specific grant from funding agencies in public, commercial, or non-profit sectors.

## Ethics approval

Not applicable.

## CRediT authorship contribution statement

**Giovanna Di Meo:** Conceptualization. **Piercarmine Panzera:** Conceptualization. **Alessandro Pasculli:** Visualization. **Francesco Paolo Prete:** Visualization. **Angela Gurrado:** Supervision. **Mario Testini:** Supervision.

## Declaration of competing interest

All authors declare that they have no conflict of interest.
